# ILC imbalance – a new piece in the gut-kidney axis puzzle

**DOI:** 10.3389/fimmu.2026.1708764

**Published:** 2026-06-02

**Authors:** Xinyin Liu, Yiwen Fang, Yicheng Xu, Xiaoran Wang, Wen Zhang

**Affiliations:** 1 Department of Traditional Chinese Medicine, Jiande First People’s Hospital, Jiande, Hangzhou, China; 2 Medical department for senior cadres, The Third Affiliated Hospital of Zhejiang Chinese Medical University, Hangzhou, China; 3 School of Bioengineering, East China University of Science and Technology, Shanghai, China; 4 Department of Nephrology, The First People’s Hospital of Hangzhou Lin’An District, Hangzhou, China; 5 Department of Nephrology, The First Affiliated Hospital of Zhejiang Chinese Medical University (Zhejiang Provincial Hospital of Chinese Medicine), Hangzhou, China

**Keywords:** acute kidney injury, aryl hydrocarbon receptor, chronic kidney disease, gut microbiota, gut-kidney axis, immunomodulation, innate lymphoid cells

## Abstract

**Background:**

The progression of chronic kidney disease (CKD) is closely linked to gut microbiota dysbiosis and the consequent accumulation of harmful microbial metabolites, forming a vicious cycle called “gut-kidney axis.” However, the specific immune mechanisms connecting these alterations in the gut milieu to renal inflammation remain poorly defined. Innate lymphoid cells (ILCs), with their tissue-resident nature, plasticity, and potential migratory capacity, are poised to be critical mediators in this process.

**Objective:**

This review aims to systematically elucidate the mechanisms by which ILCs, in response to gut-derived metabolic signals, regulate kidney disease through the gut-kidney axis.

**Methods:**

We synthesized evidence from both preclinical and clinical studies, with a focus on: ① the functions of distinct ILC subsets in kidney disease; ② the dual effects—detrimental or protective—of individual ILC subsets and their secreted factors on renal function and morphology; ③ experimental evidence linking ILC activity to the disruption of gut-kidney homeostasis.

**Conclusion:**

We propose that ILC dysregulation represents a novel immune mechanism underpinning “microbiota-gut-kidney axis” dysfunction. This review integrates existing evidence to formulate a central working model that positions the aryl hydrocarbon receptor (AHR) as a pivotal node connecting gut microbial metabolism to ILC-mediated immunity in the kidney. The trans-tissue migration of ILCs, their sensing of uremic toxins (e.g., indoxyl sulfate and kynurenine), and their synergy with adaptive immunity collectively contribute to renal injury. Future research should focus on developing novel therapeutic strategies that target the gut microbiota-ILC interaction—such as dietary interventions, probiotics, or immunomodulators—for the treatment of kidney diseases.

## 1 Introduction

Chronic kidney disease (CKD) has emerged as a global public health crisis, affecting over 850 million people worldwide (1). Beyond traditional risk factors, gut microbiota dysbiosis and the resulting disruption of microbial metabolites have been established as pivotal drivers of CKD progression, constituting a complex “gut-kidney axis” (2, 3). Specifically, patients with CKD-including those with diabetic nephropathy (DN), membranous nephropathy, and IgA nephropathy (IgAN)-exhibit reduced gut microbial diversity (4, 5), characterized by a decrease in beneficial short-chain fatty acid (SCFA)-producing bacteria and an increase in bacteria that generate uremic toxins such as indoxyl sulfate (IS) and kynurenine (Kyn) (6). The accumulation of these toxins contributes to renal damage by activating pathways involving oxidative stress, inflammasomes, among others (7). Mendelian randomization studies further support a causal relationship between specific gut bacteria and DN (8, 9).

Although the renal pathogenic role of gut microbiota and their metabolites is well-recognized, a more fundamental mechanistic question remains: How are these gut-derived biological signals sensed, integrated, and relayed by the immune system to ultimately provoke inflammation and fibrosis in the distant kidney?

The gut is not only a metabolic organ but also the body’s largest immune compartment. Therefore, dissecting the role of the local gut immune system as a mediator in gut-kidney axis is indispensable. However, research in this area remains in its early stages.

Among the diverse immune cells in the gut, innate lymphoid cells (ILCs) are considered ideal candidates for linking alterations in the gut environment to systemic immune homeostasis, owing to their tissue-residency, potent cytokine-secreting capacity, and high responsiveness to tissue microenvironmental signals (10). Recent studies revealing ILC plasticity and potential migratory capacity suggest their possible direct involvement in inter-organ immune regulation. Nevertheless, whether and how ILCs act as central immune mediators within the “gut-kidney axis” lacks systematic discussion.

This review aims to fill this knowledge gap. We will systematically dissect the multidimensional roles of ILCs within the gut-kidney axis, focusing on: ① the residency and migration of different ILC subsets in the gut and kidney; ② how ILCs respond to gut microbiota and metabolite changes associated with kidney disease; ③ how ILCs and their secreted factors influence renal outcomes. Furthermore, we propose a novel hypothetical framework for the gut-kidney axis, centered on the aryl hydrocarbon receptor (AHR)-uremic toxin combination, which connects metabolic and immune dysregulation. This work not only provides a fresh perspective on the pathogenesis of kidney disease but also lays a theoretical foundation for developing precision therapeutic strategies targeting innate immunity.

## 2 Literature search strategy

To ensure the comprehensiveness and objectivity of this review, a systematic literature search focusing on ILC-related literature was conducted. The search timeframe was set from January 2004 to October 2025, designed to encompass key studies published both before and after the formal conceptual establishment of ILCs. The primary databases searched were PubMed/MEDLINE and the Web of Science Core Collection. The search strategy integrated modern terminology (e.g., “innate lymphoid cell*”) with historical or precursor terms describing their function (e.g., “lymphoid tissue inducer”, “NKp44^+^ cell*”) to achieve comprehensive coverage of the relevant literature. Study selection was performed independently by two authors, based on pre-defined inclusion and exclusion criteria, through a process of title/abstract screening followed by full-text review. This rigorous process was implemented to provide a solid and representative literature foundation for the subsequent discussion. Additional literature was identified through targeted searches and manual curation of key references.

## 3 Biological characteristics of ILCs

ILCs were historically viewed as a class of tissue-resident innate immune cells, widely distributed throughout the body (11). ILCs predominantly reside in primary lymphoid organs (the bone marrow and thymus), secondary lymphoid organs (mesenteric lymph nodes, spleen, tonsil), and non-lymphoid tissues (e.g., the skin, liver, lungs, gastrointestinal tract, uterus, kidneys, salivary glands, and adipose tissue) (12).

Recent research, reveals these cells are not totally static entities. Their migration pathways encompass processes from generation and development to homing to specific tissues. Moreover, ILCs can migrate and respond rapidly during inflammation or infection.

### 3.1 Origin and differentiation of ILCs

Populations of lymphoid precursors identified in the fetal liver (in mice) and adult bone marrow have progressively elucidated the origin and differentiation pathway of ILCs (13). ILCs originate from common lymphoid progenitors (CLPs) (14, 15). CLPs first differentiate into bipotential CXCR6^+^ ILC/NK progenitors (αLPs). These αLPs can mature into natural killer (NK) cells or differentiate into common helper-like ILC precursors (CHILPs) (16). CHILPs give rise to common ILC progenitors (ILCPs) and lymphoid tissue inducer progenitors (LTiPs) (17). ILCPs serve as precursors for ILC1s, ILC2s, and ILC3s, while LTiPs develop into lymphoid tissue inducer (LTi) cells (17). In the adult bone marrow, most resident ILCs are functionally immature. They must migrate to specific peripheral tissues (e.g., intestine, lungs) to further differentiate into mature subsets, under the influence of local microenvironment (14, 18, 19).

Mature ILCs are distinguished from ILCPs by their subset-specific surface markers and high expression of transcription factors, with their differentiation being regulated by other specific transcription factors. Mature ILCs primarily reside in mucosal tissues, poised to rapidly respond to environmental pathogens and tissue damage without requiring specific antigen activation (20).

After 2013 (21), the new unified nomenclature proposed in 2018 categorizes ILCs into three major groups (22):

Group 1 ILCs: Include NK cells and ILC1s. ILC1s depend on T-bet, upon activation by IL-12, IL-15, or IL-18, release IFN-γ and TNF-α. Functionally analogous to helper T (Th)1 cells, providing protection against intracellular viruses and bacteria (20, 23).

Group 2 ILCs: Include ILC2s and the regulatory ILCs (ILCregs) (discovered in 2017 and characterized as Lin^-^CD45^+^CD127^+^IL-10^+^ cells) (24). ILC2s are controlled by GATA binding protein 3 (GATA3) (25) and primarily release IL-5 and IL-13 (26), upon activation by killer cell lectin-like receptor subfamily G member 1 (KLRG1) or integrin α4β7 (25), resembling Th2 cells. ILC2s play crucial roles in lung tissue homeostasis (27), helminth infection (28), and the pathophysiology of allergic diseases (29). ILCregs are found in the gut and kidneys for now. Under inflammatory conditions in the gut, they suppress the activation of ILC1s and ILC3s through IL-10 secretion and maintain/expand themselves via autocrine TGF-β1 (24, 30).

Group 3 ILCs: Include ILC3s and LTi cells. ILC3s depend on the retinoic acid-related orphan receptor γt (RORγt) and AHR. ILC3s activated by IL-1β or IL-23, release IL-17 and IL-22, similar to Th17 cells. ILC3s are involved in the formation of embryonic secondary lymphoid organs, provide defense against extracellular bacteria, and participate in mucosal immunity and tissue repair. LTi cells also depend on RORγt and secrete IL-17 and IL-22 (31).

### 3.2 Tissue localization and homing of ILCs

The tissue distribution and dynamic regulation of ILCs are influenced by multiple factors, including cytokine networks, microbial signals, and homing receptors (HRs). These factors collectively determine the localization and function of ILCs. Given the scope of this review, this section focuses on regulatory mechanisms pertinent to the gut and kidneys.

#### 3.2.1 Gut-specific ILC homing characteristics

ILC1s and ILC3s express the CCR7 receptor, which facilitates their migration from the bone marrow to the mesenteric lymph nodes (mLNs) (32, 33). Retinoic acid (RA) and vitamin A act as key inducers, upregulating the CCR9-CCL25 axis and integrin α4β7, while downregulating CCR7. CCR9 serves as a small intestine-specific homing receptor, whereas α4β7 functions as a pan-intestinal homing receptor (for both the small intestine and colon) (32). Consequently, by switching their homing receptor expression, ILC1s and ILC3s can migrate bidirectionally between the mLNs and the gut. Bone marrow-derived ILC2 precursors express CCR9, enabling RA-independent homing to the small intestine (25, 32).

Following gut homing, ILC3s and undergo further precise spatial distribution:

1. The GPR183-7α,25-dihydroxycholesterol ligand axis: This axis promotes surface expression of α4β7 integrin on ILC3s (32), and controls the distribution and accumulation of ILC3s in mLNs and PPs (34, 35). Furthermore, it drives the migration of lymphoid tissue inducer-like ILC3s (LTi-like ILC3s) toward cryptopatches (CPs) and isolated lymphoid follicles (ILFs), which is essential for lymphoid tissue formation in the colon (36).
2. CXCR6-CXCL16 chemokine axis: NKp46^+^ ILC3s constitutively express CXCR6. This receptor allows them to localize within the intestinal lamina propria villi via this axis (37).
3. Microbial metabolite influence: Commensal microbiota composition and metabolite levels vary along the GI tract, differentially regulating ILC residency. For example, elevated butyrate levels in ileal Peyer’s patches (compared to colon) suppress NKp46^+^ ILC3 residency (38).

#### 3.2.2 Composition and localization of ILCs in the kidney

In the healthy human renal cortex, helper-like ILCs (hILCs) constitute approximately 0.4% of renal lymphocytes. They predominantly exhibit a CD127^+^CD161^+^ phenotype, encompassing CRTH2^+^ ILC2s, NKp44^+^ and NKp44^-^ CRTH2^-^CD117^+^ ILC3s, and a smaller population of CRTH2^-^CD117^-^NKp44^-^ ILC1s (39). ILC2s in the kidney expressed the IL-33 receptor T1/ST2 and the IL-2 receptor high-affinity chain CD25 (39, 42).

The abundance and composition of renal ILC subsets differ notably between species (**Table 1**). In both human and mouse kidneys, ILC2s are the dominant population, yet their relative proportion diverges substantially. ILC2s account for roughly one-third of renal ILCs in humans versus approximately 80% in mice (similar to murine lungs). Conversely, ILC3s are more prominently represented in the human kidney, where NKp44^-^ and NKp44^+^ subsets together constitute over 60% of renal ILCs, compared with less than 10% in mice. ILC1s, while minor in both species, also show phenotypic divergence: human renal ILC1s are defined by CRTH2^-^CD117^-^NKp44^-^, whereas mouse ILC1s are split into NK1.1^+^ and NK1.1^-^ T-bet^+^ fractions.

**Table 1:** Comparison of ILC subset compositions in human and mouse kidneys.

Feature		Human		Mouse		References
Proportion of renal lymphocytes		~0.4%		~0.8%		(30, 39)
Major pan-markers		Lin^-^CD127^+^ CD161^+^		Lin^-^ CD127^+^		
Phenotype/median proportion among ILCs	ILC1	CRTH2^-^CD117^-^NKp44^-^	~8%	NK1.1^+^T-bet^+^	~3%	
				NK1.1^-^T-bet^+^	~2%	
	ILC2	CRTH2^+^	~33%	GATA-3^+^	~78%	
	ILC3	NKp44^+^CRTH2^-^CD117^+^	~15%	NKR^-^ RORγt^+^	~9%	
		NKp44^-^CRTH2^-^CD117^+^	~47%			
	ILCreg	IL-10^+^	~4.4%	CD90^+^IL-10^+^	~2.7%	
Key cytokine expression profile		NA		IL-13^+^	~29%	
				IL-5^+^	~26%	
				IFN-γ^+^	~14%	
				IL-17A^+^	~7%	
Localization		NA		ILC2: distributed perivascularly, in glomeruli, and in the tubulointerstitium.		(39)
				ILC3: In UUO models, distributes along renal vasculature; in LN models, localizes to renal ectopic lymphoid structures adjacent to B cells.		(40, 41)

1. Lineage (Lin) markers: Human Lin typically includes CD1a, CD3, CD4, CD11c, CD14, CD16, CD19, CD34, CD56, CD94, CD123, γδ-TCR, αβ-TCR, FcϵR1α, and BDCA1. Mouse Lin typically includes CD3, CD4, CD8, β-TCR, γδ-TCR, CD19, CD11b, CD11c, GR-1, CD49b, and Ter119.

2. IL-33R^+^ ILC2s are the predominant resident ILC subset in the kidneys of both species.

3. LN, Lupus Nephritis; NKR, Natural Killer cell Receptor; UUO, Unilateral Ureteral Obstruction.

These compositional differences carry practical implications. Since most mechanistic studies to date have been conducted in mice where ILC2s dominate more than ILC3, human ILC3s have received less attention. Moreover, information on the secreted factors and precise intrarenal distribution of human ILC subsets is still largely absent. This gap underscores the challenge of cross-species translation.

Immunohistochemical costaining for GATA3 and CD127 revealed that murine ILC2s primarily localize within the renal tubulointerstitium (adjacent to or remote from peritubular capillaries) and within glomerular tufts (39). Further precise localization studies by Cameron GJM et al. showed that renal ILC2s constitutively express higher levels of IL-5 than IL-13 under homeostatic conditions. IL-5^+^ ILC2s are broadly distributed along the kidney’s major vasculature (around the renal arteries of the cortex) (43). Their co-localization with renal dendritic cells suggests a potential pathophysiological link (43–45).

Intriguingly, pulmonary ILC2s also associate with dendritic cells and adventitial stromal cells (ASCs), and the distribution of ILC subsets is similar in mouse kidneys and lungs (43, 46). Although direct evidence is lacking, insights from pulmonary ILC2 niches may offer clues to their functions in the kidney.

ILC3s exhibit distinct localization patterns across different pathological models. In lupus nephritis (LN), ILC3s are located within renal ectopic lymphoid structures (ELS), in close proximity to B cells (41). In the unilateral ureteral obstruction (UUO) model of renal fibrosis, gut-derived ILC3s predominantly localize to perivascular fibrotic niches (40).

Notably, Cao et al. (2020) recently identified the ILCreg population — first discovered in the gut — within both human and mouse kidneys. In humans, Lin^-^CD127^+^CD161^+^IL-10^+^ ILCregs comprise ~4.4% of total renal ILCs. In mice, Lin^-^CD127^+^CD90^+^IL-10^+^ ILCregs represent ~2.7% of renal ILCs, exhibiting a surface phenotype consistent with gut ILCregs (30). This renal ILCreg population is capable of regulating innate immunity and influencing kidney diseases.

### 3.3 Plasticity of ILC subsets

Emerging evidence indicates that ILC subsets are not terminally differentiated but exhibit significant plasticity in phenotype and function. Under the influence of specific stimuli, they can transdifferentiate into other subsets.

1. ILC3 to ILC1 Conversion: *In vitro* studies demonstrate that human ILC3s, stimulated with IL-2, IL-15, and IL-23, can differentiate into CD127^+^ ILC1s. This conversion depends on the transcription factor RORγt and is enhanced by retinoic acid (47). Another study demonstrates that NKp44^+^ ILC3s derived from fetal intestine and tonsils lose c-Kit and NKp44 expression and differentiate into ILC1s upon stimulation with IL-2 and IL-12 (48). The Notch2 pathway participates in this process, along with expression of genes encoding the transcription factors T-bet, RORγt, and the AHR (49, 50).
2. ILC1 to ILC3 Conversion: CD127^+^T-bet^+^c-Kit^-^NKp44^-^ ILC1s can differentiate into NKp44^+^ ILC3s upon induction by IL-1β and IL-23, and RA amplifies this conversion (47). The IL-23-mediated interconversion between ILC1s and ILC3s may depend on the local microenvironment.
3. ILC2 to ILC1 Conversion: IL-1β serves as a key activator of ILC2s. It induces low-level expression of the transcription factor T-bet and the cytokine receptor chain IL-12Rβ2, thereby enabling ILC2s to respond to IL-12 and convert to an ILC1-like phenotype, which promotes IFN-γ expression (51, 52).
4. ILC2 to ILC3 Conversion: Under the combined action of IL-25 and Notch pathway, which upregulate RORγt expression, ILC2s can convert into ILC3 capable of secreting IL-17 and IL-13 (53, 54).

Studies within the tumor microenvironment (TME) also illustrate that ILC subsets can transdifferentiate into different subtypes (55). Current understanding of ILC plasticity requires more *in vivo* lineage-tracing data. The plasticity and diversity of ILCs underscore their potential to participate flexibly within the gut-kidney axis, and adapt to inflammatory and oxidative stress responses.

### 3.4 Migration of ILCs

During infection, injury, or inflammation within tissues, ILCs become activated and can migrate locally or across organs.

During intestinal inflammation, the GPR183-oxysterol pathway is activated (34). This mobilizes ILC3s residing in CPs, which migrate to sites of inflammation (36, 56). Huang et al. identified a migratory relationship between ILCs in the gut and lungs during helminth infection (57). Natural ILC2s primarily localized within lung tissue, potentially providing immunity against viral infections. IL-25-activated gut-derived inflammatory ILC2s express S1P receptors (S1PR1) and S1PR4, facilitating their rapid recruitment to the lungs where they mediate a transient type 2 immune response (57). Their egress from the gut via lymphatic drainage is strongly dependent on S1P1 (58, 59). The underlying mechanisms require further exploration.

Research on the dynamic migration of ILCs specifically related to the kidney remains scarce. Studies by Liang Z et al. have provided initial insights in this area (40), which we will elaborate on later in this review in the context of proposing a novel working model regarding the gut-kidney axis.

## 4 The gut-kidney axis

### 4.1 Established mechanisms of the gut-kidney axis

The gut-kidney axis refers to the bidirectional regulatory network connecting the gastrointestinal tract and kidneys, mediated by gut microbiota and their metabolites, immune cells, cytokines, and inflammatory factors.

#### 4.1.1 Metabolic regulation

Gut microbiota contribute to the pathophysiology of CKD through multiple pathways.

In healthy individuals, some species from the phylum Firmicutes, *Bifidobacterium* spp., and *Bacteroides* spp., ferment soluble dietary fibers indigestible by human enzymes, producing SCFAs to protect kidneys (60). Specifically, butyrate suppresses renal inflammation and reduces uremic toxin levels via the GPR43 signaling pathway (61). Indole-3-propionic acid (IPA) attenuates IS-induced renal oxidative stress and fibrosis by inhibiting AHR, cytochrome P450 family 1 subfamily A member 1 (CYP1A1), transforming growth factor beta 1 (TGF-β1), and monocyte chemoattractant protein-1 (MCP-1) (62).

CKD patients exhibit characteristic dysbiosis: reduced abundance of butyrate-producing bacteria (63) and increased proportions of bacteria producing urease, indole, and p-cresol-forming enzymes (63, 64). This imbalance leads to the accumulation of uremic toxins such as IS, p-cresyl sulfate (PCS), indole-3-acetic acid, trimethylamine N-oxide (TMAO), and phenylacetylglutamine (PAGln) (65). IS and PCS activate the renin-angiotensin-aldosterone system (RAAS), upregulate TGF-β expression, and induce renal tubular epithelial-mesenchymal transition (EMT) and fibrosis (66, 67). TMAO and PAGln are associated with cardiovascular complications (68, 69).

Gut dysbiosis and declining renal function form a vicious cycle. Urea excretion rely on colonic compensation (70). However, elevated blood urea concentrations cause intestinal ammonia accumulation, increased pH, and loss of tight junction proteins (e.g., claudin-1, occludin, ZO-1), resulting in increased intestinal permeability (“leaky gut”) (71, 72). This promotes translocation of endotoxins like lipopolysaccharide (LPS) and (1→3)-β-D-glucan (BG) into the circulatory system, triggering systemic inflammation (73).

#### 4.1.2 Immunoregulation

At the immunoregulatory level, soluble urokinase-type plasminogen activator receptor (suPAR) serves as a key regulatory molecule. This biomarker, secreted by immune cells and glomerular podocytes, exhibits significant positive correlations with proteinuria severity and CKD progression (74, 75). Animal studies confirm that suPAR mitigates colonic inflammatory damage by suppressing M1 macrophage polarization (76). Chronic intestinal inflammation may induce a pro-inflammatory renal microenvironment by upregulating suPAR expression (77).

Research on the gut-kidney axis often converges on the “leaky gut” mechanism, common immunoregulatory pathways remain less explored. Many immune-mediated kidney diseases, such as IgAN, ANCA-associated vasculitis (AAV), and LN, highlight distinct gut-kidney communication mechanisms centered around specific regulatory factors/cells.

IgAN: Studies indicate that overexpression of the peripheral B-cell survival factor (B cell activating factor, BAFF) leads to elevated levels of the underglycosylated, polymeric IgA containing gut microbiota-derived signals and increased IgA within the intestinal lamina propria (LP). The pathological deposition of IgA immune complexes in the glomerular mesangium (78, 79). Subsequent human studies have also confirmed that serum BAFF levels can serve as a biomarker for monitoring IgAN (80, 81).

AAV: Research reveals the small intestinal LP as a major reservoir for Th17 cells, whose homeostasis is tightly regulated by segmented filamentous bacterium (SFB) (82). Animal studies demonstrate that SFB induces host Th17 cell differentiation (83). In AAV nephritis models, Th17 cells egress from the murine gut in an S1P receptor-1 (S1P1)-dependent manner and undergo directed migration to the kidneys via the CCL20/C-C chemokine receptor type 6 (CCR6) axis. Infection with *Citrobacter rodentium* exacerbates kidney injury by amplifying intestinal Th17 cells (84).

LN: Mechanistic studies show that Lactobacillus intervention, in a sex hormone-dependent manner, reduces intestinal IL-6 levels while elevating serum and intestinal IL-10 levels, and reduces serum IgG2a (the primary immune deposit in MRL/lpr mouse kidneys). This treatment plan helps to remodel the renal Treg/Th17 balance towards to Treg population, and ameliorates renal pathology (85). Specific alterations in the abundance of the gut microbiota may play a pivotal role in the pathogenesis of lupus nephritis (86).

Although substantial evidence illustrates the impact of the gut-kidney axis on renal injury, critical gaps remain: A shared immunological framework across different kidney diseases has not been established. Current interventions often target metabolic-involved gut microbiota or single molecular entities, insufficient for addressing the systemic imbalance inherent in the gut-kidney axis.

As a key component of the innate immune system, ILCs demonstrate significant potential in linking gastrointestinal tract immune responses with renal inflammation.

## 5 ILCs: potential to bridge gaps in gut-kidney axis

The functions of ILCs across kidney disease models are summarized in **Table 2**. Most mechanistic studies in this area have relied on murine models, such as UUO, IRI, and adenine-induced CKD. Each captures only a narrow aspect of human disease. UUO and IRI, for instance, are acute and often single-hit models that do not reproduce the hemodynamic, metabolic and inflammatory drivers of clinical kidney damage. Human data, where available, remain largely correlative with mouse data. Examples include elevated circulating ILC2s in DKD and ILC3s in LN. These limitations and similarity should be kept in mind when applying these findings to clinical contexts.

**Table 2:** Comparison of ILC functions across kidney disease models.

Disease	Species	Inducing signals	Involved ILC subset	Effect (P/D)	Key findings	References
LN	Mouse Human	CSF2	ILC1	D	Renal ILC1s express CSF2, driving local macrophage expansion and thereby exacerbating kidney inflammation.	(87)
IRI-AKI	Mouse	IL-25, IL-33, IL-5	ILC2	NA	Naturally unexpanded ILC2s confer no protection against renal IRI.	(43)
IRI-AKI	Mouse	IL-25	ILC2	P	Adoptive transfer of IL-25-activated ILC2s improves renal function and mitigates histological damage.	(88)
IRI-AKI	Mouse Human	IL-33	ILC2	P	Administration of rhIL-33 or adoptive transfer of *in vitro*-expanded human ILC2s significantly alleviates renal IRI in mice.	(42)
IRI-AKI	Mouse	IL‐233	ILC2	P	IL-233 treatment increases the proportion of ST2^+^ ILC2s in the blood and kidney, and adoptive transfer of these ILC2s protects mice from IRI.	(89)
LN	Mouse	IL-33, IFN-γ, IL-27	ILC2	P	In the LN milieu, IFN-γ and IL-27 inhibit the proliferation and survival of ILC2s, while IL-33 can activate ILC2s to mitigate renal injury.	(90)
AN	Mouse	IL-33, IL-5	ILC2	P	IL-33 expands renal ILC2s, alleviating glomerulosclerosis and inflammation. ILC2-derived IL-5 induces the expansion of eosinophils in the kidney, which strongly correlates with therapeutic efficacy.	(39)
UUO-Renal fibrosis	Mouse	IL‐33	ILC2	P	UUO injury reduces renal ILC2s. Pre-treatment with IL-33 prior to UUO expands ILC2s and alleviates subsequent renal fibrosis.	(91)
Adenine-induced CKD	Mouse	IL-33, IL-13	ILC2	P	In CKD, the population of kidney-resident ILC2s is diminished. IL-33-activated renal ILC2s inhibit myofibroblast transdifferentiation by regulating Acta2 and Fn-1.	(92)
ADM/PAN induced MCD	Rat	IL‐33, IL-13	ILC2	P	IL-33 may protect against podocyte injury and reduce proteinuria by promoting the secretion of IL-13 from ILC2s.	(93)
Obesity-associated T2DN	Mouse	IL-233	ILC2	P	IL-233 may protect against T2DN by expanding ILC2s, which recruit eosinophils and macrophages, thereby alleviating proteinuria and glomerular mesangial expansion.	(94)
NA	Mouse	IL‐33, IL-4, IL-5, IL-13	ILC2	D	HIF activation downregulates the IL-33 receptor (ST2L) on ILC2s, inhibiting their production of IL-5/IL-13 and subsequent M2 macrophage polarization, which may contribute to an anti-fibrotic effect.	(95)
IgAN	Mouse	IL-33	ILC2	D	IL-33-activated ILC2s exacerbate proteinuria, glomerulosclerosis, tubulointerstitial damage, and the deposition of IgA and C3 in glomeruli.	(96)
DKD	Human	IL-4, IL-5, IL-13	ILC2	D	In DKD patients, the proportion of ILC2s in peripheral blood and the levels of IL-4, IL-5, and IL-13 are significantly elevated, which may exacerbate renal fibrosis partly through the TGF-β1 signaling pathway.	(97)
crystal-induced kidney fibrosis	Mouse	IL-23, IL- 1β	ILC3	D	ILC3s exacerbate renal inflammation and fibrosis through inflammasome activation, a process regulated by IL-1β and IL-23.	(98)
FAN/UUO-Renal fibrosis	Mouse Human	IL-17A, IL-23	ILC3	D	Gut-derived CXCR6+ ILC3s migrate to the kidney under the guidance of CXCL16, driving renal interstitial fibrosis by upregulating PD-1 expression and producing large amounts of IL-17A.	(40)
LN	Human Mouse	CXCL16	ILC3	D	Gut-derived ILC3s migrate to the kidney and localize adjacent to B cells within ELS, exacerbating autoimmunity and renal injury, and directly activate B cells to differentiate into plasma cells.	(41)
LN	Mouse	IL-22	ILC3	D	ILC3s produce IL-22, promoting macrophage infiltration in the kidney and aggravating nephropathy.	(99)
IRI-AKI	Mouse Human	IL‐2C	ILCreg	P	ILCregs proliferated by IL‐2C alleviate IRI‐AKI.	(30)

ADM, Adriamycin; AKI, Acute Kidney Injury; AN, Adriamycin Nephropathy; CKD, Chronic Kidney Disease; CSF2, Colony Stimulating Factor 2; DKD, Diabetic Kidney Disease; ELS, Ectopic Lymphoid Structures; FAN, Folic Acid Nephropathy; HIF, Hypoxia-Inducible Factor; IgAN, Immunoglobulin A Nephropathy; IL-2C, IL-2 Complex; IRI, Ischemia-Reperfusion Injury; LN, Lupus Nephritis; MCD, Minimal Change Disease; PAN, Puromycin Aminonucleoside; PD-1, Programmed Cell Death Protein 1; rhIL-33, Recombinant Human Interleukin-33; ST2L, Suppression of Tumorigenicity 2; TGF-β1, Transforming Growth Factor Beta 1; T2DN, Type 2 Diabetic Nephropathy; UUO, Unilateral Ureteral Obstruction. D, Detrimental; NA, Not Applicable; P, Protective.

### 5.1 Regulatory roles of ILC3s and their secreted factors

#### 5.1.1 Direct stimulation by renal ILC3s

Kidney-resident ILC3s can directly stimulate aberrant accumulation of mononuclear phagocytes within the kidney. This activates the chronic inflammasome pathway, leading to TGF-β production, which in turn stimulates fibroblasts to produce collagen. Ultimately, this cascade exacerbates renal inflammation and fibrosis. Multiple cytokines orchestrate this process: IL-23 sustains ILC3 survival, IL-1β promotes ILC3 proliferation and enhances its pro-fibrotic capacity, and IL-18 drives the differentiation of the NKp46^+^ ILC3 subset (98). In LN, ILC3s localize in proximity to B cells within renal ELS, where they directly activate B cells to differentiate into plasma cells and produce antibodies, a process mediated by the Delta-like 1 (DLL1)/Notch pathway (41).

#### 5.1.2 The dual role of IL-22 in kidney injury

IL-22, a core secretory factor of ILC3s, exhibits a dual role in kidney injury.

In a mouse model of acute kidney injury (AKI) induced by IRI, recombinant mouse IL-22 binds to IL-22R1 on renal proximal tubular epithelial cells. IL-22 activates the STAT3 and Akt pathways, up-regulates the anti-apoptotic gene Bcl-2, and suppresses the pro-apoptotic gene Bad, thereby reducing inflammation and tubular cell damage (100). Activated STAT3 in endothelial cells forms a positive feedback loop sustaining endogenous IL-22 expression, reducing oxidative stress and macrophage infiltration, and limiting proximal tubular damage in IRI (101). Another research revealed IL-22 from proximal tubules prevents the overactivation of DNA damage response (DDR) effector proteins (e.g., p53), confining cisplatin and aristolochic acid-induced renal DDR within a pro-repair threshold, thus preventing pathological tubular cell death (102).

Conversely, in CKD models induced by angiotensin II (Ang II), recombinant mouse IL-22 promotes renal interstitial inflammatory infiltration, fibrosis, and hypertension via the STAT3 pathway. The anti-IL-22 neutralizing monoclonal antibody and the special STAT3 pathway inhibitor S31–201 can mitigate this damage (103). A similar result was obtained in a study using MRL/lpr mice. Binding of ILC3-derived IL-22 to its receptor (IL-22R) on renal epithelial cells activated the STAT3 signaling pathway, which enhanced the secretion of chemokines, promoted macrophage infiltration into the kidney, and thereby exacerbated LN (99).

Of note, many functional studies of IL-22 have employed recombinant protein, which provides valuable insight into downstream signaling but does not inform the question of cellular source. Among studies examining endogenous IL-22, the source has rarely been pinpointed. Where it has—as in the Ang II model—the pathogenic IL-22 was predominantly Th22-derived rather than ILC3-derived. The divergent renal effects of IL-22 may thus depend critically on cellular origin, yet this variable remains largely unexplored.

#### 5.1.3 ILC3s and gut barrier dysfunction

As key components of the intestinal mucosal immune system, ILC3s contribute to gastrointestinal epithelial barrier integrity and microbial homeostasis under physiological conditions by producing appropriate levels of IL-22, IL-17, IFN-γ, and granulocyte-macrophage colony-stimulating factor (GM-CSF) (104).

However, microbial infections can break this protective role. Studies by Ferguson et al. have highlighted the functional role of ILCs in SIV (Simian Immunodeficiency Virus)-infected macaque model, wherein they substitute for CD4+ T lymphocytes in cytokine secretion to maintain intestinal homeostasis (105). Xu et al., using an SIV-infected macaque model, demonstrated that persistent microbial translocation induces dual expression of TLR2 and TLR4 on intestinal ILC3s and subsequently activates Toll-like receptor 2 (TLR2) (recognizing lipoteichoic acid) and/or Toll-like receptor 4 (TLR4) (recognizing LPS) pathways. This activation triggers apoptosis of ILC3s and exacerbates structural and functional damage to the gut-associated lymphoid tissue (GALT) (106). These studies confirmed the association between ILC3 dysfunction and the “leaky gut” state, suggesting that impaired ILC3 function may serve as a critical immunological symbol of abnormal gut barrier permeability.

Furthermore, NCR^+^ ILC3s undergo conversion toward ILC1s under IL-12 stimulation from CD14^+^ or CD130^+^ dendritic cells (DCs). This occurs alongside functional impairment of the remaining NCR^-^ ILC3 or NCR^+^ ILC3 populations, collectively leading to overexpression of IL-22, IL-17, and IFN-γ. IL-17 recruits neutrophils which disrupt E-cadherin and junctional adhesion molecule-like (JAML) molecules, increases epithelial permeability and contributes to the progression of intestinal inflammation (104).

#### 5.1.4 ILC3 migration pathway in the gut-kidney axis and pro-fibrotic mechanism

Murine studies have revealed that gut-derived CXCR6^+^ ILC3s can undergo rapid inter-organ migration via the CXCL16-CXCR6 axis. Liang Z et al. confirmed through immunofluorescence and flow cytometry that ILC3 accumulation in fibrotic kidneys stems more significantly from extrarerenal sources than from local proliferation. Following renal tubular injury, released CXCL16 drives the accumulation of CXCR6^+^ ILC3s within the kidney. Once in the kidney, these ILC3s exhibit increased expression of programmed death-1 (PD-1) and IL-17A. IL-17A directly activates myofibroblasts. PD-1 amplifies IL-23-induced JAK2/STAT3/RORγt/IL-17A signaling in ILC3s by competitively binding IL-23R to inhibit endocytosis, further amplifying the fibrotic response (40). Li F. et al. confirmed the existence of the gut–kidney ILC3 migration pathway in LN. They further indicated that a systemic elevation of ILC3s, particularly within the kidneys, is highly correlated with disease severity in both human and murine LN (41).

However, direct functional validation of the CXCR6/CXCL16 axis in human kidney disease remains limited. The human LN data demonstrate a correlation, but whether this axis drives ILC3 migration in the human kidney has yet to be established.

#### 5.1.5 Hypothesis: AHR as a central player in ILC3-related gut-kidney axis

AHR is a ligand-dependent nuclear transcription factor that binds diverse endogenous and exogenous ligands. AHR resides in the cytoplasm without ligand. AHR exists in many immune cells (e.g., T cells, MΦ, DC, ILCs) (107).

AHR is critically important for intestinal barrier integrity. It influences the development and maintenance of RORγt^+^ ILCs (108), assists in IL-22 production (109), and is essential for intestinal lymphoid follicle formation (110). Notably, among ILCs, RORγt^+^ expression is almost exclusively a surface marker for LTi cells and NKp44^+^/NKp44^-^ ILC3s, underscoring the vital role of ILC3s in gut homeostasis (22).

During kidney disease progression, the accumulation of high levels of uremic toxins in the circulation significantly impacts both the gut and kidneys. The relevance of AHR to the gut–kidney axis lies in the fact that the uremic toxins indoxyl 3-sulfate (I3S) and Kyn are potent endogenous AHR ligands (111, 112). We therefore hypothesize that uremic toxin-driven AHR activation in intestinal ILC3s may disrupt gut barrier homeostasis—a concept expanded in the working model below. However, the binding affinity, dose-response effects, and relative priority of IS and Kyn compared to other AHR ligands require further research.

There is some evidence supporting inter-organ migration of ILC3s between the gut and kidney. Liang Z et al. demonstrated that gut-derived CXCR6^+^ ILC3s are recruited to the kidneys under the influence of CXCL16 released following renal tubular injury (40).

Upon entering renal microenvironment, ILC3s undergo further activation, characterized by a marked upregulation of PD-1 expression on their cell surface. On one hand, PD-1, by competitively binding and impeding the endocytosis of the IL-23 receptor (IL-23R), enhances the responsiveness of ILC3s to IL-23. This mechanism sustains and amplifies the downstream JAK2/STAT3/RORγt signaling axis, ultimately leading to a substantial increase in the capacity of ILC3s to secrete IL-17A (40). The overproduced IL-17A acts on the IL-17 receptor (IL-17R) on the surface of renal tubular epithelial cells, inducing the expression of cytokine and chemokine genes, which robustly drives local renal inflammation and promotes renal fibrosis (40, 113, 114).

On the other hand, indoleamine 2,3-dioxygenase (IDO)1, a key enzyme regulating AhR and tryptophan metabolism, may also play a role in mediating resistance to anti-PD-1 therapy (115, 116). This suggests that PD-1 activation could lead to local enrichment of Kyn in the kidney via an IDO1-dependent mechanism.

Additionally, other studies have observed increased AHR activation in the renal tubules of mice with CKD and AKI (117). Research by Xie et al. (118) suggests that renal AHR can interact with peroxisome proliferator-activated receptor gamma coactivator 1-alpha (PGC1α, a key regulator of mitochondrial biogenesis) (119) and promote its ubiquitin-mediated degradation via its E3 ubiquitin ligase activity. The elevation and activation of AHR by IS may suppress mitochondrial biogenesis, thereby accelerating renal aging and fibrosis.

**Figure 1:**
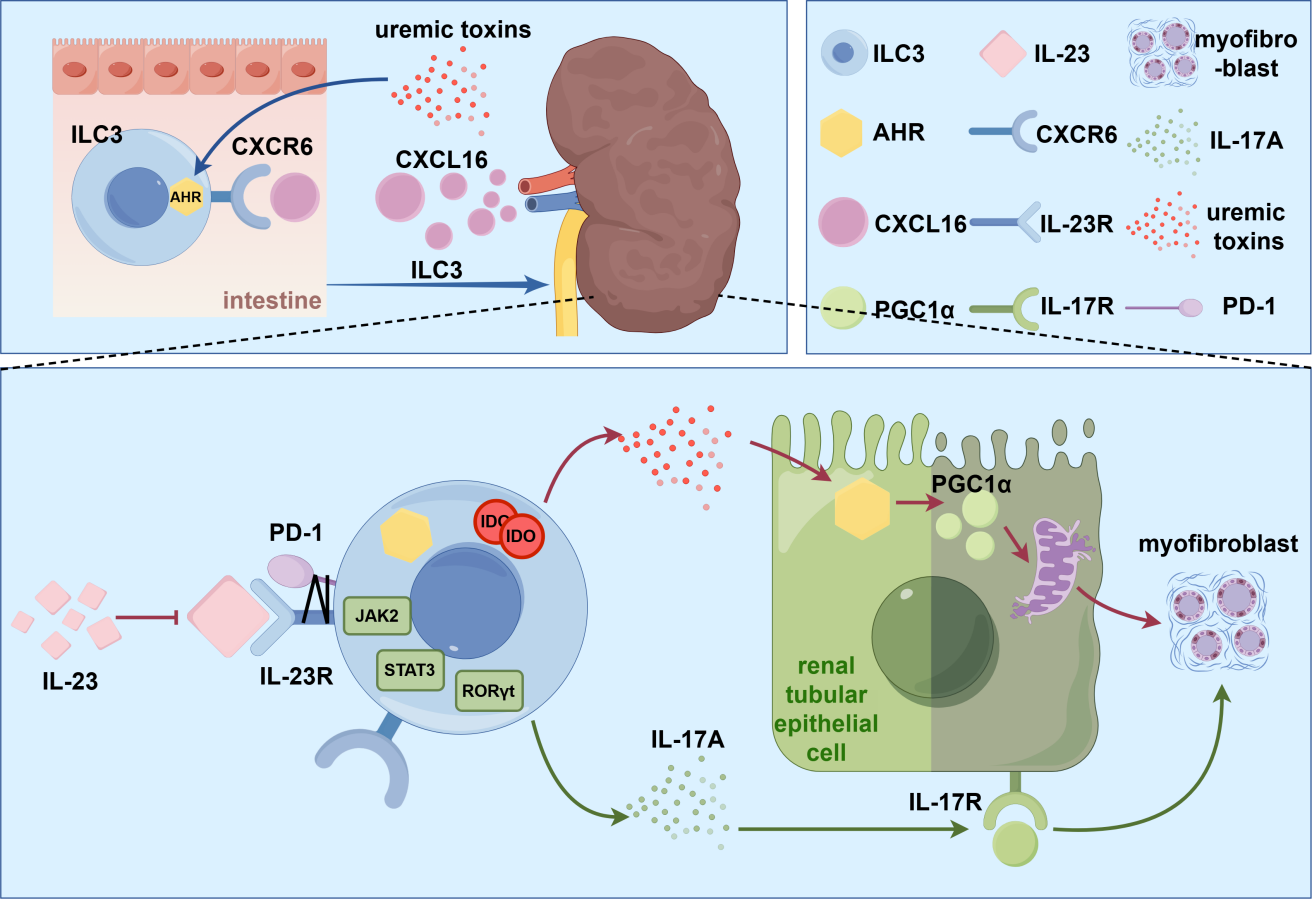
The working model of ILC3 involvement in the gut-kidney axis. AHR, Aryl Hydrocarbon Receptor; CXCL16, C-X-C Motif Chemokine Ligand 16; CXCR6, C-X-C Motif Chemokine Receptor 6; ILC, Innate Lymphoid Cell; PD-1, Programmed Death-1; PGC1α, peroxisome proliferator-activated receptor gamma coactivator 1-alpha.

We propose a working model (**Figure 1**) in which the following events may constitute a pathological cascade linking gut ILC3s to chronic renal fibrosis, though several steps remain to be validated directly.

In this model, systemically accumulated uremic toxins (such as IS and Kyn) could act as endogenous ligands to activate the AHR within intestinal ILC3s. It is plausible that aberrant, sustained AHR activation not only disrupts intestinal barrier homeostasis but also primes ILC3s toward a pathological, pro-migratory phenotype, potentially including upregulation of CXCR6 (direct evidence linking uremic toxin-AHR binding to CXCR6 induction in ILC3s is currently lacking).

Studies have demonstrated that gut-derived ILC3s are specifically recruited to the kidney via the CXCR6/CXCL16 axis. Within the local renal microenvironment, the expression of PD-1 on their cell membrane is significantly upregulated. Based on findings from Liang Z et al., this PD-1 upregulation could amplify IL-23R/JAK2/STAT3/RORγt signaling by competitively inhibiting IL-23R endocytosis, thereby markedly increasing IL-17A secretion capacity. Our model extends this by speculating that the PD-1 upregulation could induce the expression of IDO in ILC3s, catalyzing the conversion of tryptophan to Kyn, which could locally enrich AHR ligands within the kidney—thereby establishing a feedback loop that remains to be tested.

IL-17A acts on renal tubular epithelial cells, driving a robust local inflammatory response. Concurrently, the same accumulated uremic toxin ligands may activate the intrinsic AHR within the tubular cells. The activated AHR has been shown, through its E3 ubiquitin ligase activity, to promote the ubiquitination and degradation of peroxisome proliferator-activated receptor gamma coactivator 1-alpha (PGC1α)—a key regulator of mitochondrial biogenesis—leading to mitochondrial dysfunction. We reason that the IL-17A-driven inflammation and the cellular metabolic crisis resulting from impairment of the AHR-PGC1α axis act synergistically to accelerate the progression of renal interstitial fibrosis. Notably, the link between ILC3-derived IL-17A and renal tubular AHR-mediated metabolic dysfunction has not been experimentally interrogated and represents a core prediction of our model.

Based on this mechanism, dietary intervention to competitively antagonize the aberrant activation of intestinal ILC3s by uremic toxins, thereby improving gut-kidney axis homeostasis, emerges as a promising novel therapeutic strategy.

Certain studies suggest that dietary AHR ligands may ameliorate autoimmune-associated inflammation. Cruciferous vegetables (e.g., broccoli, cauliflower, cabbage) yield AHR ligands like indole-3-carbinol (I3C) and diindolylmethane (DIM) through enzymatic breakdown and gastric acid conversion (120, 121).

However, it is crucial to emphasize that the final outcome of AHR modulation is jointly determined by the intervention route, site of activation, and type of disease.

In the experimental autoimmune encephalomyelitis model, administration of I3C/DIM via injection achieves body-wide AHR activation, effectively promoting Tregs and suppressing Th17 cells, thereby treating the disease (122). Conversely, in the type 1 diabetes (NOD) model, dietary supplementation with I3C primarily leads to localized AHR activation within the gut. This failed to induce beneficial systemic immunomodulation and instead exacerbated pancreatitis (123).

Different delivery methods (e.g., injection, oral) dictate the distinct distribution and metabolism of AHR ligands within the body, resulting in markedly different immunological and pathological outcomes. In the context of CKD, a critical question arises: can specifically designed dietary AhR ligands, delivered via specific intervention routes, selectively antagonize the harmful signaling from uremic toxins at the gut level while avoiding adverse immune consequences, thereby constituting a safe therapeutic strategy? This warrants further research.

In summary, ILC2s exhibit a triad of characteristics: tissue residency, migration within the gut and between the gut and kidneys, and AHR-centric bidirectional immunoregulatory functions. This cell population is thus highly implicated in the acute and chronic pathophysiological processes of the gut-kidney axis.

### 5.2 Regulatory roles of ILC2s and their secreted factors

#### 5.2.1 ILC2s in AKI

Helper-like ILCs constitute 0.5%-3% of leukocytes in mouse and human kidneys, with ILC2s being the predominant subset (39). Consequently, most research focused on ILC2s. Renal IRI and cisplatin-induced nephrotoxicity are the most common AKI models.

Interestingly, natural unexpanded ILC2s are not considered protective against renal IRI and may exist only as a minor population within the kidney for reasons that remain unclear (43).

However, ILC2s activated by various cytokines can confer protection in IRI mouse models. IL-25-Activated ILC2s reduce renal tubular cell damage and improve kidney function in mice. The underlying mechanism may involve the production of IL-4, IL-5, IL-13 by ILC2s and the stimulation of M2 macrophages in kidney (88). Cao et al. demonstrated that IL-33 activates and expands renal ILC2s, or adoptive transfer of human ILC2s ameliorates renal injury in IRI humanized mice (42). This protective effect in IRI appears amphiregulin (Areg)-dependent (124). Areg, a key factor produced by IL-33-activated ILC2s, is also proven to restore epithelial integrity and homeostasis during gut injury (125). IL-233, a fusion cytokine of IL-2 and IL-33, expands the proportion of ST2^+^ ILC2s in the blood and kidney, contributing to the amelioration of AKI injury (89). Mouse experiments by Nagashima R. et al. demonstrated that IL-33 pre-treatment-induced renal ILC2s confer protection against UUO-induced renal fibrosis (91). Conversely, in a subsequent study, the same research team found that ILC2s may promote renal fibrosis via the Hypoxia-inducible factor-1α (HIF-1α)/ILC2s/M2 macrophage axis (95). A. Akcay et al. found that administration of high-dose IL-33 exacerbates cisplatin-induced AKI in a CD4^+^ T cell-dependent manner (126).

These divergent findings likely reflect the context-dependent nature of ILC2 function. The net renal effect of ILC2 activation appears to be shaped by several interacting variables, including the insult type, the intensity and timing of upstream cytokine signals, and the microenvironment. In the setting of acute injury with moderate, timely IL-33 signaling, ILC2s may favor an Areg-mediated repair program; with sustained or excessive stimulation, however, a shift toward IL-13/HIF-1α-driven pathways may lead to promote fibrosis.

Therefore, future research must more precisely define the activation switch of ILC2s, their crosstalk with other immune cells, and the microenvironmental signals that dictate their function within specific disease contexts.

#### 5.2.2 ILC2s in CKD

ILC2s show promising therapeutic potential in CKD, demonstrating broad renoprotective functions.

1. Cytokine-mediated protection. In adenine-induced CKD, IL-33-activated ILC2s preferentially produced IL-13 over IL-5, accompanied by reduced GATA3 expression. This activation suppressed myofibroblast transdifferentiation via Acta2 and Fn-1, attenuating fibrosis (92). IL-33-mediated ILC2 activation also limited injury in AN, LN, and MCD models, where IL-13 secretion contributed to protective mechanism (39, 90, 93).
2. Th2 immune responses. IL-25-driven ILC2 activation may induce Th2 responses and M2 macrophage accumulation, attenuating injury in adriamycin nephropathy (AN) mice (127). Similarly, activation of ILC2s and Tregs by IL-233 elicits a Th2-like response, promoting the accumulation of alternatively activated macrophages and eosinophils. This cascade protects genetically obese mice from type 2 diabetic nephropathy (T2DN), alleviating renal inflammation, glomerular hypertrophy, and mesangial expansion (128).
3. Prolonged Tissue Residence: ILC2s exhibit a remarkably long half-life. Daily administration of recombinant IL-33 (400 ng, i.p., 4 doses) for 7 days to C57BL/6 mice induces kidney-resident ILC2 expansion lasting up to 8 weeks (39). This persistence suggests their potential for long-term maintenance therapy in clinical settings.

However, ILC2-based therapy remains unresolved questions.

1. IL-5 produced by ILC2s induces eosinophil expansion in the kidneys of AN mice, correlating strongly with therapeutic efficacy (39). However, eosinophils are often pathogenic in interstitial nephritis and autoimmune diseases (129).
2. ILC2s have been shown to promote tissue fibrosis in the liver and lungs via induction of type 2 immunity (130, 131). ILC2s may contribute to renal fibrosis in diabetic kidney disease (DKD) partly through the TGF-β1 signaling pathway (97).
3. One study found that IL-33 appears to promote IgA production through the expansion of ILC2s, thereby exacerbating IgAN in mice (96).
4. Two key negative regulators of ILC2s—IFN-γ and IL-27—form a complex cross-regulatory network with activators like IL-25 and IL-33 (90). The precise pathophysiological roles and balance of this network in kidney diseases remain poorly understood and warrant further exploration.

These questions further illustrate the context-dependent nature of ILC2 function introduced in Section 5.2.1. Even within the same disease spectrum, the net renal effect of ILC2 activation can diverge. In the T2DN model, controlled, therapeutically-timed IL-233 administration drives a protective Th2 response. In progressive DKD, sustained endogenous activation under chronic metabolic stress may instead favor TGF-β1-dependent pro-fibrotic pathways. This underscores that the outcome is shaped less by the disease than by the intensity, duration, and context of ILC2 stimulation.

#### 5.2.3 ILC2s and the gut-kidney axis

Within the stomach, the vast majority of ILCs are ILC2s, with ILC1s being rare and RORγt^-^ ILC3s virtually absent. Their homeostasis and effector functions are regulated by the local commensal microbiota (132). Notably, some meta-analyses have found a significantly lower prevalence of *Helicobacter pylori (H. pylori)* infection in CKD patients compared to controls, the reasons for which remain unclear (133, 134). Conversely, *H. pylori*-infected individuals exhibit increased susceptibility to autoimmune disorders and immune-mediated nephropathies (e.g., IgAN, systemic lupus erythematosus (SLE)) (135, 136).

The synergistic interplay between ILC2s and B cells reveals novel immunopathological insights. *H. pylori* disrupts gastrointestinal homeostasis (137). Following *H. pylori* infection, the stomach induces the production of interleukin-7 (IL-7) and IL-33, which in turn trigger ILC2 activation and expansion. ILC2-derived IL-5 promotes IgA production. This IgA coats gastric bacteria in both specific pathogen-free (SPF) and *H. pylori-*infected mice, thereby protecting the gastric microenvironment (132).

Although studies have confirmed that ILC2s can support B cells in the spleen and lungs (138, 139), and that *H. pylori* infection specifically activates gastric ILC2s, the mechanism by which gastric ILC2s might remotely influence renal B cells remains to be elucidated. We propose two plausible pathways:

1. Systemic dissemination of cytokines. Activated gastric ILC2s produce large amounts of cytokines such as IL-5 and IL-13, which may enter the circulation. These cytokines could provide sustained signals for the differentiation, activation, survival, and antibody production of kidney-resident B cells, thereby exacerbating local autoantibody production or immune complex deposition.
2. Altered B-cell homing and migration. B cells activated in the gastric microenvironment by ILC2−derived signals may alter their homing properties and migrate to the kidney, where they could participate in pathological processes—a mechanism analogous to that observed in the gut−lung axis.

Current research predominantly indicates that activation of renal ILC2s exerts a protective effect within the kidney itself. Whether deeper inter-organ communication and feedback coordination exist between gastrointestinal and renal ILC2 populations requires further investigation.

Future studies could be conducted to test this hypothesis as follows:

1. In patient cohorts with B cell−mediated nephropathies (e.g., IgAN, LN), examine the correlation between *H. pylori* infection status and the frequency/activation of peripheral ILC2s, serum levels of IL−5/IL−13, and renal pathological features.
2. In animal models of H. pylori infection, neutralize ILC2−derived cytokines such as IL−5 and IL−13 to assess whether this specifically attenuates B cell−related kidney pathology.
3. Employ cell−tracking techniques to trace whether H. pylori−activated gastric ILC2s migrate to the kidney and contribute to local inflammation.

### 5.3 Detrimental role of ILC1s in LN

Research on ILC1s in the kidney is limited and primarily associated with SLE. Biniaris-Georgallis et al. demonstrated that NKp46+ ILC1s play a pivotal role in initiating renal inflammation (87). Blocking the NKp46-mediated ILC response protected the renal parenchyma and reduced podocyte damage. Notably, serum anti-dsDNA antibody titers were unaffected, indicating that autoantibody levels do not correlate with renal injury; rather, the titers is linked to the activation of NKp46+ ILC1s. This research group proposed that in LN, NKp46+ ILC1s within the renal parenchyma are activated during autoimmunity via binding to unidentified NKp46+ ligands, then they produce GM-CSF or colony stimulating factor 2 (CSF2). Spatially targeted interactions between ILC1s and CSF2R^+^ monocyte-derived macrophages drive macrophage population expansion, thereby affecting renal inflammation. This shows ILC1 cell activation as a key driver of kidney damage in LN.

## 6 Conclusion

The recognition that ILC imbalance underpins gut-kidney axis dysregulation opens a new therapeutic perspective. While most existing evidence derives from preclinical studies, scattered clinical findings have begun to substantiate the impact of ILCs in human kidney diseases. For instance, the accumulation of ILC3s in the kidneys of patients with lupus nephritis has been detected and correlates with disease activity (41), while the functional state of ILC2s may be associated with fibrotic progression in diabetic kidney disease (97). Consequently, studying key ILC features—including their migration, immune crosstalk, and AHR-mediated responses—offers a promising path toward novel strategies for kidney disease.

We propose that future research efforts prioritize two key translational directions, with an emphasis on expanding human-based studies: first, validating ILCs or their characteristic cytokine profiles as biomarkers for predicting disease progression in large patient cohorts; and second, exploring precision interventions that target the “microbiota-ILC-kidney” axis—such as dietary metabolites (e.g., AHR ligands), probiotics, or targeted immunomodulators—followed by interventional clinical trials to evaluate their efficacy and safety. Translating this mechanistic framework into clinical practice is essential for realizing precise immunotherapeutic approaches for kidney diseases.

## Glossary

AAV: ANCA-associated vasculitis
AHR: aryl hydrocarbon receptor
AKI: acute kidney injury
Ang II: angiotensin II
AN: adriamycin nephropathy
Areg: amphiregulin
ASCs: adventitial stromal cells
BAFF: B cell activating factor
BG: (1→3)-β-D-glucan
CHILPs: common helper-like ILC precursors
CKD: chronic kidney disease
CLPs: common lymphoid progenitors
CPs: cryptopatches
CYP1A1: cytochrome P450 family 1 subfamily A member 1
DCs: dendritic cells
DDR: DNA damage response
DIM: diindolylmethane
DN: diabetic nephropathy
ELS: ectopic lymphoid structures
EMT: epithelial-mesenchymal transition
GALT: gut-associated lymphoid tissue
GATA3: GATA binding protein 3
GM-CSF: granulocyte-macrophage colony-stimulating factor
HIF-1α: hypoxia-inducible factor-1α
H. pylori: Helicobacter pylori
hILCs: helper-like innate lymphoid cells
HRs: homing receptors
I3C: indole-3-carbinol
I3S: indoxyl 3-sulfate
IDO: indoleamine 2,3-dioxygenase
IgAN: IgA nephropathy
ILCs: innate lymphoid cells
ILCPs: common ILC progenitors
ILCregs: regulatory innate lymphoid cells
ILFs: isolated lymphoid follicles
IPA: indole-3-propionic acid
IRI: ischemia/reperfusion injury
IS: indoxyl sulfate
KLRG1: killer cell lectin-like receptor subfamily G member 1
Kyn: kynurenine
LN: lupus nephritis
LPS: lipopolysaccharide
LTi cells: lymphoid tissue inducer cells
LTiPs: lymphoid tissue inducer progenitors
MCP-1: monocyte chemoattractant protein-1
mLNs: mesenteric lymph nodes
PAGln: phenylacetylglutamine
PCS: p-cresyl sulfate
PGC1α: peroxisome proliferator-activated receptor gamma coactivator 1-alpha
RA: retinoic acid
RAAS: renin-angiotensin-aldosterone system
RORγt: retinoic acid-related orphan receptor γt
SCFAs: short-chain fatty acids
SFB: segmented filamentous bacterium
SIV: Simian Immunodeficiency Virus
SLE: systemic lupus erythematosus
suPAR: soluble urokinase-type plasminogen activator receptor
T2DN: type 2 diabetic nephropathy
TGF- β1: transforming growth factor beta 1
TMAO: trimethylamine N-oxide
TME: tumor microenvironment
TLR2: Toll-like receptor 2
TLR4: Toll-like receptor 4
UUO: unilateral ureteral obstruction

## References

[1] Jager KJ Kovesdy C Langham R Rosenberg M Jha V Zoccali C . A single number for advocacy and communication-worldwide more than 850 million individuals have kidney diseases. Nephrol Dial Transplant. (2019) 34(11):1803–5. doi: 10.1093/ndt/gfz174. PMID: 31566230

[2] Tsuji K Uchida N Nakanoh H Fukushima K Haraguchi S Kitamura S . The gut–kidney axis in chronic kidney diseases. Diagnostics. (2024) 15:21. doi: 10.3390/diagnostics15010021. PMID: 39795549 PMC11719742

[3] Tao P Huo J Chen L . Bibliometric analysis of the relationship between gut microbiota and chronic kidney disease from 2001–2022. Integr Med Nephrol Andrology. (2024) 11:e00017. doi: 10.1097/imna-d-23-00017. PMID: 42160389

[4] Wang X Liu X Gong F Jiang Y Zhang C Zhou W . Targeting gut microbiota for diabetic nephropathy treatment: Probiotics, dietary interventions, and fecal microbiota transplantation. Front Endocrinol. (2025) 16:1621968. doi: 10.3389/fendo.2025.1621968. PMID: 40661744 PMC12256261

[5] Zhang L Hu L Tan L Zhang Z Chen M Gan W . The dysbiosis of gut microbiota and dysregulation of metabolites in iga nephropathy and membranous nephropathy. Front Med. (2025) 12:1618947. doi: 10.3389/fmed.2025.1618947. PMID: 40740937 PMC12307357

[6] Corradi V Caprara C Barzon E Mattarollo C Zanetti F Ferrari F . A possible role of p-cresyl sulfate and indoxyl sulfate as biomarkers in the prediction of renal function according to the gfr (g) categories. Integr Med Nephrol Andrology. (2024) 11:e24-00002. doi: 10.1097/imna-d-24-00002. PMID: 42160389

[7] Jin Y Zhang S-J Zhuang S Li P Miao H Zhao Y-Y . Microbiota-gut-kidney axis in health and renal disease. Int J Biol Sci. (2026) 22:750. doi: 10.7150/ijbs.125140. PMID: 41522358 PMC12781074

[8] Cao B-N Zhang C-Y Wang Z Wang Y-X . Causal relationship between 412 gut microbiota, 1,400 blood metabolites, and diabetic nephropathy: A randomized mendelian study. Front Endocrinol. (2025) 15:1450428. doi: 10.3389/fendo.2024.1450428. PMID: 39897958 PMC11782027

[9] Song S Ning L Yu J . Elucidating the causal relationship between gut microbiota, metabolites, and diabetic nephropathy in european patients: Revelations from genome-wide bidirectional mendelian randomization analysis. Front Endocrinol. (2025) 15:1391891. doi: 10.3389/fendo.2024.1391891. PMID: 39845884 PMC11750691

[10] Elemam NM Ramakrishnan RK Hundt JE Halwani R Maghazachi AA Hamid Q . Innate lymphoid cells and natural killer cells in bacterial infections: Function, dysregulation, and therapeutic targets. Front Cell Infect Microbiol. (2021) 11:733564. doi: 10.3389/fcimb.2021.733564. PMID: 34804991 PMC8602108

[11] Alisjahbana A Mohammad I Gao Y Evren E Ringqvist E Willinger T . Human macrophages and innate lymphoid cells: Tissue-resident innate immunity in humanized mice. Biochem Pharmacol. (2020) 174:113672. doi: 10.1016/j.bcp.2019.113672. PMID: 31634458

[12] Meininger I Carrasco A Rao A Soini T Kokkinou E Mjösberg J . Tissue-specific features of innate lymphoid cells. Trends Immunol. (2020) 41:902–17. doi: 10.1016/j.it.2020.08.009. PMID: 32917510

[13] Wang X Li J Rebuffet L Cheng M Bao B Chen Y . Innate lymphoid cells originate from fetal liver–derived tissue-resident progenitors. Sci Immunol. (2025) 10:eadu7962. doi: 10.1126/sciimmunol.adu7962. PMID: 40644510

[14] Constantinides MG McDonald BD Verhoef PA Bendelac A . A committed precursor to innate lymphoid cells. Nature. (2014) 508:397–401. doi: 10.1038/nature13047. PMID: 24509713 PMC4003507

[15] Harly C Cam M Kaye J Bhandoola A . Development and differentiation of early innate lymphoid progenitors. J Exp Med. (2018) 215:249–62. doi: 10.1084/jem.20170832. PMID: 29183988 PMC5748853

[16] Kim CH Hashimoto-Hill S Kim M . Migration and tissue tropism of innate lymphoid cells. Trends Immunol. (2016) 37:68–79. doi: 10.1016/j.it.2015.11.003. PMID: 26708278 PMC4744800

[17] Bartemes KR Kita H . Roles of innate lymphoid cells (ilcs) in allergic diseases: The 10-year anniversary for ilc2s. J Allergy Clin Immunol. (2021) 147:1531–47. doi: 10.1016/j.jaci.2021.03.015. PMID: 33965091 PMC8114584

[18] Klose CS Flach M Möhle L Rogell L Hoyler T Ebert K . Differentiation of type 1 ilcs from a common progenitor to all helper-like innate lymphoid cell lineages. Cell. (2014) 157:340–56. doi: 10.1016/j.cell.2014.03.030. PMID: 24725403

[19] Possot C Schmutz S Chea S Boucontet L Louise A Cumano A . Notch signaling is necessary for adult, but not fetal, development of rorγt+ innate lymphoid cells. Nat Immunol. (2011) 12:949–58. doi: 10.1038/ni.2105. PMID: 21909092

[20] Kortekaas Krohn I Shikhagaie MM Golebski K Bernink J Breynaert C Creyns B . Emerging roles of innate lymphoid cells in inflammatory diseases: Clinical implications. Allergy. (2018) 73:837–50. doi: 10.1111/all.13340. PMID: 29069535

[21] Spits H Artis D Colonna M Diefenbach A Di Santo JP Eberl G . Innate lymphoid cells—a proposal for uniform nomenclature. Nat Rev Immunol. (2013) 13:145–9. doi: 10.1038/nri3365. PMID: 23348417

[22] Vivier E Artis D Colonna M Diefenbach A Di Santo JP Eberl G . Innate lymphoid cells: 10 years on. Cell. (2018) 174:1054–66. doi: 10.1016/j.cell.2018.07.017. PMID: 30142344

[23] Taggenbrock RL van Gisbergen KP . Ilc1: Development, maturation, and transcriptional regulation. Eur J Immunol. (2023) 53:2149435. doi: 10.1002/eji.202149435. PMID: 36408791 PMC10099236

[24] Wang S Xia P Chen Y Qu Y Xiong Z Ye B . Regulatory innate lymphoid cells control innate intestinal inflammation. Cell. (2017) 171:201–16. doi: 10.1016/j.cell.2017.07.027. PMID: 28844693

[25] Hoyler T Klose CS Souabni A Turqueti-Neves A Pfeifer D Rawlins EL . The transcription factor gata-3 controls cell fate and maintenance of type 2 innate lymphoid cells. Immunity. (2012) 37:634–48. doi: 10.1016/j.immuni.2012.06.020. PMID: 23063333 PMC3662874

[26] Maspero J Adir Y Al-Ahmad M Celis-Preciado CA Colodenco FD Giavina-Bianchi P . Type 2 inflammation in asthma and other airway diseases. ERJ Open Res. (2022) 8(3):00576–2021. doi: 10.1183/23120541.00576-2021. PMID: 35923421 PMC9339769

[27] Verma M Can UI Reinhardt RL . Ilc2 diversity, location, and function in pulmonary disease. Immunol Rev. (2025) 332:e70036. doi: 10.1111/imr.70036. PMID: 40454563 PMC12128188

[28] Wang Y Zhang X Liu S Gu Z Sun Z Zang Y . Bi-directional communication between intrinsic enteric neurons and ilc2s inhibits host defense against helminth infection. Immunity. (2025) 58:465–80. doi: 10.1016/j.immuni.2025.01.004. PMID: 39889704

[29] Pasha MA Patel G Hopp R Yang Q . Role of innate lymphoid cells in allergic diseases. Allergy Asthma Proc. (2019) 40(3):138–45. doi: 10.2500/aap.2019.40.4217. PMID: 31018888 PMC6500789

[30] Cao Q Wang R Wang Y Niu Z Chen T Wang C . Regulatory innate lymphoid cells suppress innate immunity and reduce renal ischemia/reperfusion injury. Kidney Int. (2020) 97:130–42. doi: 10.1016/j.kint.2019.07.019. PMID: 31685310

[31] Pelletier A Stockmann C . The metabolic basis of ilc plasticity. Front Immunol. (2022) 13:858051. doi: 10.3389/fimmu.2022.858051. PMID: 35572512 PMC9099248

[32] Kim MH Taparowsky EJ Kim CH . Retinoic acid differentially regulates the migration of innate lymphoid cell subsets to the gut. Immunity. (2015) 43:107–19. doi: 10.1016/j.immuni.2015.06.009. PMID: 26141583 PMC4511719

[33] Mackley EC Houston S Marriott CL Halford EE Lucas B Cerovic V . Ccr7-dependent trafficking of rorγ+ ilcs creates a unique microenvironment within mucosal draining lymph nodes. Nat Commun. (2015) 6:5862. doi: 10.1038/ncomms6862. PMID: 25575242 PMC4354100

[34] Willinger T . Oxysterols in intestinal immunity and inflammation. J Intern Med. (2019) 285:367–80. doi: 10.1111/joim.12855. PMID: 30478861 PMC7379495

[35] Chu C Moriyama S Li Z Zhou L Flamar A-L Klose CS . Anti-microbial functions of group 3 innate lymphoid cells in gut-associated lymphoid tissues are regulated by g-protein-coupled receptor 183. Cell Rep. (2018) 23:3750–8. doi: 10.1016/j.celrep.2018.05.099. PMID: 29949760 PMC6209103

[36] Emgård J Kammoun H García-Cassani B Chesné J Parigi SM Jacob J-M . Oxysterol sensing through the receptor gpr183 promotes the lymphoid-tissue-inducing function of innate lymphoid cells and colonic inflammation. Immunity. (2018) 48:120–32. doi: 10.1016/j.immuni.2017.11.020. PMID: 29343433 PMC5772175

[37] Satoh-Takayama N Serafini N Verrier T Rekiki A Renauld J-C Frankel G . The chemokine receptor cxcr6 controls the functional topography of interleukin-22 producing intestinal innate lymphoid cells. Immunity. (2014) 41:776–88. doi: 10.1016/j.immuni.2014.10.007. PMID: 25456160

[38] Kim S-H Cho B-H Kiyono H Jang Y-S . Microbiota-derived butyrate suppresses group 3 innate lymphoid cells in terminal ileal peyer’s patches. Sci Rep. (2017) 7:3980. doi: 10.1038/s41598-017-02729-6. PMID: 28638068 PMC5479798

[39] Riedel J-H Becker M Kopp K Düster M Brix SR Meyer-Schwesinger C . Il-33–mediated expansion of type 2 innate lymphoid cells protects from progressive glomerulosclerosis. J Am Soc Nephrol. (2017) 28:2068–80. doi: 10.1681/ASN.2016080877. PMID: 28154198 PMC5491284

[40] Liang Z Tang Z Zhu C Li F Chen S Han X . Intestinal cxcr6+ ilc3s migrate to the kidney and exacerbate renal fibrosis via il-23 receptor signaling enhanced by pd-1 expression. Immunity. (2024) 57:1306–23. doi: 10.1016/j.immuni.2024.05.004. PMID: 38815582 PMC11539045

[41] Li F Liang Z Zhong H Hu X Tang Z Zhu C . Group 3 innate lymphoid cells exacerbate lupus nephritis by promoting b cell activation in kidney ectopic lymphoid structures. Adv Sci. (2023) 10:2302804. doi: 10.1002/advs.202302804. PMID: 37915129 PMC10724443

[42] Cao Q Wang Y Niu Z Wang C Wang R Zhang Z . Potentiating tissue-resident type 2 innate lymphoid cells by il-33 to prevent renal ischemia-reperfusion injury. J Am Soc Nephrol. (2018) 29:961–76. doi: 10.1681/ASN.2017070774. PMID: 29295873 PMC5827602

[43] Cameron GJ Cautivo KM Loering S Jiang SH Deshpande AV Foster PS . Group 2 innate lymphoid cells are redundant in experimental renal ischemia-reperfusion injury. Front Immunol. (2019) 10:826. doi: 10.3389/fimmu.2019.00826. PMID: 31057549 PMC6477147

[44] Dahlgren MW Molofsky AB . Adventitial cuffs: Regional hubs for tissue immunity. Trends Immunol. (2019) 40:877–87. doi: 10.1016/j.it.2019.08.002. PMID: 31522963 PMC6823140

[45] Becker M Gnirck A-C Turner J-E . Innate lymphoid cells in renal inflammation. Front Immunol. (2020) 11:72. doi: 10.3389/fimmu.2020.00072. PMID: 32063905 PMC7000421

[46] Dahlgren MW Jones SW Cautivo KM Dubinin A Ortiz-Carpena JF Farhat S . Adventitial stromal cells define group 2 innate lymphoid cell tissue niches. Immunity. (2019) 50:707–22. doi: 10.1016/j.immuni.2019.02.002. PMID: 30824323 PMC6553479

[47] Bernink JH Krabbendam L Germar K de Jong E Gronke K Kofoed-Nielsen M . Interleukin-12 and-23 control plasticity of cd127+ group 1 and group 3 innate lymphoid cells in the intestinal lamina propria. Immunity. (2015) 43:146–59. doi: 10.1016/j.immuni.2015.06.019. PMID: 26187413

[48] Bernink JH Peters CP Munneke M Te Velde AA Meijer SL Weijer K . Human type 1 innate lymphoid cells accumulate in inflamed mucosal tissues. Nat Immunol. (2013) 14:221–9. doi: 10.1038/ni.2534. PMID: 23334791

[49] Chea S Perchet T Petit M Verrier T Guy-Grand D Banchi E-G . Notch signaling in group 3 innate lymphoid cells modulates their plasticity. Sci Signal. (2016) 9:ra45-ra. doi: 10.1126/scisignal.aaf2223. PMID: 27141929

[50] Viant C Rankin LC Girard-Madoux MJ Seillet C Shi W Smyth MJ . Transforming growth factor–β and notch ligands act as opposing environmental cues in regulating the plasticity of type 3 innate lymphoid cells. Sci Signal. (2016) 9:ra46-ra. doi: 10.1126/scisignal.aaf2176. PMID: 27141930

[51] Lim AI Menegatti S Bustamante J Le Bourhis L Allez M Rogge L . Il-12 drives functional plasticity of human group 2 innate lymphoid cells. J Exp Med. (2016) 213:569–83. doi: 10.1084/jem.20151750. PMID: 26976630 PMC4821648

[52] Ohne Y Silver JS Thompson-Snipes L Collet MA Blanck JP Cantarel BL . Il-1 is a critical regulator of group 2 innate lymphoid cell function and plasticity. Nat Immunol. (2016) 17:646–55. doi: 10.1038/ni.3447. PMID: 27111142

[53] Huang Y Guo L Qiu J Chen X Hu-Li J Siebenlist U . Il-25-responsive, lineage-negative klrg1hi cells are multipotential ‘inflammatory’ type 2 innate lymphoid cells. Nat Immunol. (2015) 16:161–9. doi: 10.1038/ni.3078. PMID: 25531830 PMC4297567

[54] Zhang K Xu X Pasha MA Siebel CW Costello A Haczku A . Cutting edge: Notch signaling promotes the plasticity of group-2 innate lymphoid cells. J Immunol. (2017) 198:1798–803. doi: 10.4049/jimmunol.1601421. PMID: 28115527 PMC5321819

[55] Heinrich B Korangy F . Plasticity of innate lymphoid cells in cancer. Front Immunol. (2022) 13. doi: 10.3389/fimmu-13-886520. PMID: 35663967 PMC9160464

[56] Pearson C Thornton EE McKenzie B Schaupp A-L Huskens N Griseri T . Ilc3 gm-csf production and mobilisation orchestrate acute intestinal inflammation. Elife. (2016) 5:e10066. doi: 10.7554/eLife.10066. PMID: 26780670 PMC4733039

[57] Huang Y Mao K Chen X Sun M Kawabe T Li W . S1p-dependent interorgan trafficking of group 2 innate lymphoid cells supports host defense. Science. (2018) 359:114–9. doi: 10.1126/science.aam5809. PMID: 29302015 PMC6956613

[58] Gasteiger G Fan X Dikiy S Lee SY Rudensky AY . Tissue residency of innate lymphoid cells in lymphoid and nonlymphoid organs. Science. (2015) 350:981–5. doi: 10.1126/science.aac9593. PMID: 26472762 PMC4720139

[59] Germain RN Huang Y . Ilc2s—resident lymphocytes pre-adapted to a specific tissue or migratory effectors that adapt to where they move? Curr Opin Immunol. (2019) 56:76–81. doi: 10.1016/j.coi.2018.11.001. PMID: 30472437 PMC6945789

[60] Guan Z-W Yu E-Z Feng Q . Soluble dietary fiber, one of the most important nutrients for the gut microbiota. Molecules. (2021) 26:6802. doi: 10.3390/molecules26226802. PMID: 34833893 PMC8624670

[61] Li H-B Xu M-L Xu X-D Tang Y-Y Jiang H-L Li L . Faecalibacterium prausnitzii attenuates ckd via butyrate-renal gpr43 axis. Circ Res. (2022) 131:e120–34. doi: 10.1002/advs.202302804. PMID: 36164984 PMC9588706

[62] Yisireyili M Takeshita K Saito S Murohara T Niwa T . Indole-3-propionic acid suppresses indoxyl sulfate-induced expression of fibrotic and inflammatory genes in proximal tubular cells. Nagoya J Med Sci. (2017) 79:477. doi: 10.18999/nagjms.79.4.477. PMID: 29238104 PMC5719207

[63] Liu P Yang J Chen Y Zhu Y Tang Y Xu X . Alterations of gut microbiota and metabolome in early chronic kidney disease patients complicated with hyperuricemia. Heliyon. (2023) 9(9):e20328. doi: 10.1016/j.heliyon.2023.e20328. PMID: 37809388 PMC10560056

[64] Peters BA Qi Q Usyk M Daviglus ML Cai J Franceschini N . Association of the gut microbiome with kidney function and damage in the hispanic community health study/study of latinos (hchs/sol). Gut Microbes. (2023) 15:2186685. doi: 10.1080/19490976.2023.2186685. PMID: 36882941 PMC10012940

[65] Khiabani SA Asgharzadeh M Kafil HS . Chronic kidney disease and gut microbiota. Heliyon. (2023) 9(8):e18991. doi: 10.1016/j.heliyon.2023.e18991. PMID: 37609403 PMC10440536

[66] Sun C-Y Chang S-C Wu M-S . Uremic toxins induce kidney fibrosis by activating intrarenal renin–angiotensin–aldosterone system associated epithelial-to-mesenchymal transition. PloS One. (2012) 7:e34026. doi: 10.1371/journal.pone.0034026. PMID: 22479508 PMC3316590

[67] Chen J-H Chao C-T Huang J-W Hung K-Y Liu S-H Tarng D-C . Early elimination of uremic toxin ameliorates aki-to-ckd transition. Clin Sci. (2021) 135:2643–58. doi: 10.1042/CS20210858. PMID: 34796904

[68] Nemet I Saha PP Gupta N Zhu W Romano KA Skye SM . A cardiovascular disease-linked gut microbial metabolite acts via adrenergic receptors. Cell. (2020) 180:862–77:e22. doi: 10.1016/j.cell.2020.02.016. PMID: 32142679 PMC7402401

[69] Amaritei O Mierlan OL Dinu CA Chiscop I Matei MN Gutu C . Tmao and cardiovascular disease: Exploring its potential as a biomarker. Med (Mex). (2025) 61:1767. doi: 10.3390/medicina61101767. PMID: 41155754 PMC12565763

[70] Hatch M Vaziri N . Enhanced enteric excretion of urate in rats with chronic renal failure. Clin Sci. (1994) 86:511–6. doi: 10.1042/cs0860511. PMID: 8033505

[71] Vaziri ND Yuan J Norris K . Role of urea in intestinal barrier dysfunction and disruption of epithelial tight junction in chronic kidney disease. Am J Nephrol. (2013) 37:1–6. doi: 10.1159/000345969. PMID: 23258127 PMC3686571

[72] Kuo WT Odenwald MA Turner JR Zuo L . Tight junction proteins occludin and zo‐1 as regulators of epithelial proliferation and survival. Ann N Y Acad Sci. (2022) 1514:21–33. doi: 10.1111/nyas.14798. PMID: 35580994 PMC9427709

[73] Tungsanga S Udompornpitak K Worasilchai J Ratana-Aneckchai T Wannigama DL Katavetin P . Candida administration in 5/6 nephrectomized mice enhanced fibrosis in internal organs: An impact of lipopolysaccharide and (1→ 3)-β-d-glucan from leaky gut. Int J Mol Sci. (2022) 23:15987. doi: 10.3390/ijms232415987. PMID: 36555628 PMC9784901

[74] Wei C Möller CC Altintas MM Li J Schwarz K Zacchigna S . Modification of kidney barrier function by the urokinase receptor. Nat Med. (2008) 14:55–63. doi: 10.1038/nm1696. PMID: 18084301

[75] Reiser J Hayek SS Sever S . The role of supar and related proteins in kidney, heart diseases, and diabetes. J Clin Invest. (2026) 136(1):e197141. doi: 10.1172/JCI197141. PMID: 41480757 PMC12721894

[76] Mansouri P Mansouri P Behmard E Najafipour S Kouhpayeh A Farjadfar A . Novel targets for mucosal healing in inflammatory bowel disease therapy. Int Immunopharmacol. (2025) 144:113544. doi: 10.1016/j.intimp.2024.113544. PMID: 39571265

[77] Liu X Wang X Zhang P Fang Y Liu Y Ding Y . Intestinal homeostasis in the gut-lung-kidney axis: A prospective therapeutic target in immune-related chronic kidney diseases. Front Immunol. (2023) 14:1266792. doi: 10.3389/fimmu.2023.1266792. PMID: 38022571 PMC10646503

[78] McCarthy DD Chiu S Gao Y Summers-deLuca LE Gommerman JL . Baff induces a hyper-iga syndrome in the intestinal lamina propria concomitant with iga deposition in the kidney independent of light. Cell Immunol. (2006) 241:85–94. doi: 10.1016/j.cellimm.2006.08.002. PMID: 16987502

[79] McCarthy DD Kujawa J Wilson C Papandile A Poreci U Porfilio EA . Mice overexpressing baff develop a commensal flora–dependent, iga-associated nephropathy. J Clin Invest. (2011) 121:3991–4002. doi: 10.1172/JCI45563. PMID: 21881212 PMC3195458

[80] Xin G Shi W Xu L-X Su Y Yan L-J Li K-S . Serum baff is elevated in patients with iga nephropathy and associated with clinical and histopathological features. J Nephrol. (2012) 26:683–90. doi: 10.5301/jn.5000218. PMID: 23042433

[81] Zheng N Fan J Wang B Wang D Feng P Yang Q . Expression profile of baff in peripheral blood from patients of iga nephropathy: Correlation with clinical features and streptococcus pyogenes infection. Mol Med Rep. (2017) 15:1925–35. doi: 10.3892/mmr.2017.6190. PMID: 28260100

[82] Ivanov II Atarashi K Manel N Brodie EL Shima T Karaoz U . Induction of intestinal th17 cells by segmented filamentous bacteria. Cell. (2009) 139:485–98. doi: 10.1016/j.cell.2009.09.033. PMID: 19836068 PMC2796826

[83] Yang Y Torchinsky MB Gobert M Xiong H Xu M Linehan JL . Focused specificity of intestinal th17 cells towards commensal bacterial antigens. Nature. (2014) 510:152–6. doi: 10.1038/nature13279. PMID: 24739972 PMC4128479

[84] Krebs CF Paust H-J Krohn S Koyro T Brix SR Riedel J-H . Autoimmune renal disease is exacerbated by s1p-receptor-1-dependent intestinal th17 cell migration to the kidney. Immunity. (2016) 45:1078–92. doi: 10.1016/j.immuni.2016.10.020. PMID: 27851911 PMC6381450

[85] Mu Q Zhang H Liao X Lin K Liu H Edwards MR . Control of lupus nephritis by changes of gut microbiota. Microbiome. (2017) 5:73. doi: 10.1186/s40168-017-0300-8. PMID: 28697806 PMC5505136

[86] Wang A Zhao J Qin Y Zhang Y Xing Y Wang Y . Alterations of the gut microbiota in the lupus nephritis: A systematic review. Ren Fail. (2023) 45:2285877. doi: 10.1080/0886022X.2023.2285877. PMID: 37994423 PMC11001323

[87] Biniaris-Georgallis S-I Aschman T Stergioula K Schreiber F Jafari V Taranko A . Amplification of autoimmune organ damage by nkp46-activated ilc1s. Nature. (2024) 634:952–60. doi: 10.1038/s41586-024-07907-x. PMID: 39137897

[88] Huang Q Niu Z Tan J Yang J Liu Y Ma H . Il-25 elicits innate lymphoid cells and multipotent progenitor type 2 cells that reduce renal ischemic/reperfusion injury. J Am Soc Nephrol. (2015) 26:2199–211. doi: 10.1681/ASN.2014050479. PMID: 25556172 PMC4552110

[89] Stremska ME Jose S Sabapathy V Huang L Bajwa A Kinsey GR . Il233, a novel il-2 and il-33 hybrid cytokine, ameliorates renal injury. J Am Soc Nephrol. (2017) 28:2681–93. doi: 10.1681/ASN.2016121272. PMID: 28539382 PMC5576940

[90] Düster M Becker M Gnirck AC Wunderlich M Panzer U Turner JE . T cell‐derived ifn‐γ downregulates protective group 2 innate lymphoid cells in murine lupus erythematosus. Eur J Immunol. (2018) 48:1364–75. doi: 10.1002/eji.201747303. PMID: 29671873

[91] Nagashima R Ishikawa H Kuno Y Kohda C Iyoda M . Il-33 attenuates renal fibrosis via group2 innate lymphoid cells. Cytokine. (2022) 157:155963. doi: 10.1016/j.cyto.2022.155963. PMID: 35868116

[92] Nagashima R Ishikawa H Kuno Y Kohda C Eshima K Iyoda M . Group2 innate lymphoid cells ameliorate renal fibrosis and dysfunction associated with adenine-induced ckd. Cell Immunol. (2024) 401:104828. doi: 10.1016/j.cellimm.2024.104828. PMID: 38759328

[93] Liu C Liu L Huang Y Shi R Wu Y Ismail IHB . Contribution of il-33/ilc2-mediated th2 cytokines during the progression of minimal change disease. Int Immunopharmacol. (2023) 114:109493. doi: 10.1016/j.intimp.2022.109493. PMID: 36527879

[94] Sabapathy V Stremska ME Mohammad S Corey RL Sharma PR Sharma R . Novel immunomodulatory cytokine regulates inflammation, diabetes, and obesity to protect from diabetic nephropathy. Front Pharmacol. (2019) 10:572. doi: 10.3389/fphar.2019.00572. PMID: 31191312 PMC6540785

[95] Nagashima R Ishikawa H Kuno Y Kohda C Iyoda M . Hif-phd inhibitor regulates the function of group2 innate lymphoid cells and polarization of m2 macrophages. Sci Rep. (2023) 13:1867. doi: 10.1038/s41598-023-29161-3. PMID: 36725898 PMC9892566

[96] Wang YM Shaw K Zhang GY Chung EY Hu M Cao Q . Interleukin-33 exacerbates iga glomerulonephritis in transgenic mice overexpressing b cell activating factor. J Am Soc Nephrol. (2022) 33:966–84. doi: 10.1681/ASN.2021081145. PMID: 35387873 PMC9063894

[97] Liu C Qin L Ding J Zhou L Gao C Zhang T . Group 2 innate lymphoid cells participate in renal fibrosis in diabetic kidney disease partly via tgf‐β1 signal pathway. J Diabetes Res. (2019) 2019:8512028. doi: 10.1155/2019/8512028. PMID: 31355294 PMC6636594

[98] Frasconi TM Kurts C Dhana E Kaiser R Reichelt M Lukacs-Kornek V . Renal il-23–dependent type 3 innate lymphoid cells link crystal-induced intrarenal inflammasome activation with kidney fibrosis. J Immunol. (2024) 213:865–75. doi: 10.4049/jimmunol.2400041. PMID: 39072698 PMC11372247

[99] Hu L Hu J Chen L Zhang Y Wang Q Yang X . Interleukin-22 from type 3 innate lymphoid cells aggravates lupus nephritis by promoting macrophage infiltration in lupus-prone mice. Front Immunol. (2021) 12:584414. doi: 10.3389/fimmu.2021.584414. PMID: 33717066 PMC7953152

[100] Xu M-J Feng D Wang H Guan Y Yan X Gao B . Il-22 ameliorates renal ischemia-reperfusion injury by targeting proximal tubule epithelium. J Am Soc Nephrol. (2014) 25:967–77. doi: 10.1681/ASN.2013060611. PMID: 24459233 PMC4005304

[101] Dube S Matam T Yen J Mang HE Dagher PC Hato T . Endothelial stat3 modulates protective mechanisms in a mouse ischemia‐reperfusion model of acute kidney injury. J Immunol Res. (2017) 2017:4609502. doi: 10.1155/2017/4609502. PMID: 29181415 PMC5664346

[102] Taguchi K Sugahara S Elias BC Pabla NS Canaud G Brooks CR . Il-22 is secreted by proximal tubule cells and regulates DNA damage response and cell death in acute kidney injury. Kidney Int. (2024) 105:99–114. doi: 10.1016/j.kint.2023.09.020. PMID: 38054920 PMC11068062

[103] Ye J Ji Q Liu J Liu L Huang Y Shi Y . Interleukin 22 promotes blood pressure elevation and endothelial dysfunction in angiotensin ii–treated mice. J Am Heart Assoc. (2017) 6:e005875. doi: 10.1161/JAHA.117.005875. PMID: 28974499 PMC5721831

[104] Zeng B Shi S Ashworth G Dong C Liu J Xing F . Ilc3 function as a double-edged sword in inflammatory bowel diseases. Cell Death Dis. (2019) 10:315. doi: 10.1038/s41419-019-1540-2. PMID: 30962426 PMC6453898

[105] Ferguson N Cogswell A Barker E . Contribution of innate lymphoid cells in supplementing cytokines produced by cd4+ t cells during acute and chronic siv infection of the colon. AIDS Res Hum Retrovir. (2022) 38:709–25. doi: 10.1089/AID.2022.0007. PMID: 35459417 PMC9514600

[106] Xu H Wang X Lackner AA Veazey RS . Type 3 innate lymphoid cell depletion is mediated by tlrs in lymphoid tissues of simian immunodeficiency virus–infected macaques. FASEB J. (2015) 29:5072. doi: 10.1096/fj.15-276477. PMID: 26283536 PMC4653054

[107] Shinde R McGaha TL . The aryl hydrocarbon receptor: Connecting immunity to the microenvironment. Trends Immunol. (2018) 39:1005–20. doi: 10.1016/j.it.2018.10.010. PMID: 30409559 PMC7182078

[108] Qiu J Zhou L . Aryl hydrocarbon receptor promotes RORγt^+^ group 3 ILCs and controls intestinal immunity and inflammation. Semin Immunopathol. (2013) 35(6):657–70. doi: 10.1007/s00281-013-0393-5 PMC3797199 23975386

[109] Kang H Chen Z Wang B Chen Z . The ahr/il-22 axis in chronic gut inflammation: Unraveling mechanisms and therapeutic prospects. Front Immunol. (2025) 16:1668173. doi: 10.3389/fimmu.2025.1668173. PMID: 41019044 PMC12463905

[110] Kiss EA Vonarbourg C Kopfmann S Hobeika E Finke D Esser C . Natural aryl hydrocarbon receptor ligands control organogenesis of intestinal lymphoid follicles. Science. (2011) 334:1561–5. doi: 10.1126/science.1214914. PMID: 22033518

[111] Campesato LF Budhu S Tchaicha J Weng C-H Gigoux M Cohen IJ . Blockade of the ahr restricts a treg-macrophage suppressive axis induced by l-kynurenine. Nat Commun. (2020) 11:4011. doi: 10.1038/s41467-020-17750-z. PMID: 32782249 PMC7419300

[112] Sadeghi Shermeh A Royzman D Kuhnt C Draßner C Stich L Steinkasserer A . Differential modulation of dendritic cell biology by endogenous and exogenous aryl hydrocarbon receptor ligands. Int J Mol Sci. (2023) 24:7801. doi: 10.3390/ijms24097801. PMID: 37175508 PMC10177790

[113] Ramani K Biswas PS . Emerging roles of the th17/il-17-axis in glomerulonephritis. Cytokine. (2016) 77:238–44. doi: 10.1016/j.cyto.2015.07.029. PMID: 26440138

[114] Liao W Fan R Du Y Wang H Yang Y Tian Y . Nafamostat mesilate attenuates renal fibrosis by suppressing the il-17 signaling pathway. Front Pharmacol. (2025) 16:1648623. doi: 10.3389/fphar.2025.1648623. PMID: 41244830 PMC12615172

[115] Zhang Y Chen Y Xia J Li L Chang L Luo H . Rifaximin ameliorates influenza a virus infection-induced lung barrier damage by regulating gut microbiota. Appl Microbiol Biotechnol. (2024) 108:469. doi: 10.1007/s00253-024-13280-6. PMID: 39298023 PMC11413070

[116] Botticelli A Cerbelli B Lionetto L Zizzari I Salati M Pisano A . Can ido activity predict primary resistance to anti-pd-1 treatment in nsclc? J Transl Med. (2018) 16:219. doi: 10.1186/s12967-018-1595-3. PMID: 30081936 PMC6080500

[117] Walker JA Richards S Belghasem ME Arinze N Yoo SB Tashjian JY . Temporal and tissue-specific activation of aryl hydrocarbon receptor in discrete mouse models of kidney disease. Kidney Int. (2020) 97:538–50. doi: 10.1016/j.kint.2019.09.029. PMID: 31932072 PMC9721455

[118] Xie H Yang N Lu L Sun X Li J Wang X . Uremic toxin receptor ahr facilitates renal senescence and fibrosis via suppressing mitochondrial biogenesis. Adv Sci. (2024) 11:2402066. doi: 10.1002/advs.202402066. PMID: 38940381 PMC11434102

[119] Fontecha-Barriuso M Martin-Sanchez D Martinez-Moreno JM Monsalve M Ramos AM Sanchez-Niño MD . The role of pgc-1α and mitochondrial biogenesis in kidney diseases. Biomolecules. (2020) 10:347. doi: 10.3390/biom10020347. PMID: 32102312 PMC7072614

[120] Ito S Chen C Satoh J Yim S Gonzalez FJ . Dietary phytochemicals regulate whole-body cyp1a1 expression through an arylhydrocarbon receptor nuclear translocator–dependent system in gut. J Clin Invest. (2007) 117:1940–50. doi: 10.1172/JCI31647. PMID: 17607366 PMC1890999

[121] Bjeldanes LF Kim J-Y Grose KR Bartholomew JC Bradfield CA . Aromatic hydrocarbon responsiveness-receptor agonists generated from indole-3-carbinol *in vitro* and *in vivo*: Comparisons with 2, 3, 7, 8-tetrachlorodibenzo-p-dioxin. Proc Natl Acad Sci. (1991) 88:9543–7. doi: 10.1073/pnas.88.21.9543. PMID: 1658785 PMC52754

[122] Rouse M Singh NP Nagarkatti PS Nagarkatti M . Indoles mitigate the development of experimental autoimmune encephalomyelitis by induction of reciprocal differentiation of regulatory t cells and th17 cells. Br J Pharmacol. (2013) 169:1305–21. doi: 10.1111/bph.12205. PMID: 23586923 PMC3831710

[123] Kahalehili HM Newman NK Pennington JM Kolluri SK Kerkvliet NI Shulzhenko N . Dietary indole-3-carbinol activates ahr in the gut, alters th17-microbe interactions, and exacerbates insulitis in nod mice. Front Immunol. (2021) 11:606441. doi: 10.3389/fimmu.2020.606441. PMID: 33552063 PMC7858653

[124] Wang R Wang Y Harris DC Cao Q . Innate lymphoid cells in kidney diseases. Kidney Int. (2021) 99:1077–87. doi: 10.1016/j.kint.2020.11.023. PMID: 33387602

[125] Monticelli LA Osborne LC Noti M Tran SV Zaiss DM Artis D . Il-33 promotes an innate immune pathway of intestinal tissue protection dependent on amphiregulin–egfr interactions. Proc Natl Acad Sci. (2015) 112:10762–7. doi: 10.1073/pnas.1509070112. PMID: 26243875 PMC4553775

[126] Akcay A Nguyen Q He Z Turkmen K Lee DW Hernando AA . Il-33 exacerbates acute kidney injury. J Am Soc Nephrol. (2011) 22:2057–67. doi: 10.1681/ASN.2010091011. PMID: 21949094 PMC3279998

[127] Cao Q Wang C Zheng D Wang Y Lee VW Wang YM . Il-25 induces m2 macrophages and reduces renal injury in proteinuric kidney disease. J Am Soc Nephrol. (2011) 22:1229–39. doi: 10.1681/ASN.2010070693. PMID: 21719780 PMC3137571

[128] He QD Huang YP Zhu LB Shen JC Lian LY Zhang Y . Difference of liver and kidney metabolic profiling in chronic atrophic gastritis rats between acupuncture and moxibustion treatment. Evid Based Complement Alternat Med. (2018) 2018:6030929. doi: 10.1155/2018/6030929. PMID: 30310411 PMC6166372

[129] Wu EY Hernandez ML Jennette JC Falk RJ . Eosinophilic granulomatosis with polyangiitis: Clinical pathology conference and review. J Allergy Clin Immunology: In Pract. (2018) 6:1496–504. doi: 10.1016/j.jaip.2018.07.001. PMID: 30197069

[130] Liu M-K Tang J-J Li H Chen X-Y Cai J-L Lin G-Y . Artemisitene ameliorates carbon tetrachloride-induced liver fibrosis by inhibiting nlrp3 inflammasome activation and modulating immune responses. Int Immunopharmacol. (2025) 146:113818. doi: 10.1016/j.intimp.2024.113818. PMID: 39681062

[131] Fonseca W Lukacs NW Elesela S Malinczak C-A . Role of ilc2 in viral-induced lung pathogenesis. Front Immunol. (2021) 12:675169. doi: 10.3389/fimmu.2021.675169. PMID: 33953732 PMC8092393

[132] Satoh-Takayama N Kato T Motomura Y Kageyama T Taguchi-Atarashi N Kinoshita-Daitoku R . Bacteria-induced group 2 innate lymphoid cells in the stomach provide immune protection through induction of iga. Immunity. (2020) 52:635–49. doi: 10.1016/j.immuni.2020.03.002. PMID: 32240600

[133] Shin SP Bang CS Lee JJ Baik GH . Helicobacter pylori infection in patients with chronic kidney disease: A systematic review and meta-analysis. Gut Liver. (2019) 13:628. doi: 10.5009/gnl18517. PMID: 30970438 PMC6860029

[134] Hata K Koyama T Ozaki E Kuriyama N Mizuno S Matsui D . Assessing the Relationship between Helicobacter pylori and Chronic Kidney Disease. Healthcare (Basel). (2021) 9(2):162. doi: 10.3390/healthcare9020162 PMC7913305 33546229

[135] Liu X-Z Zhang Y-M Jia N-Y Zhang H . Helicobacter pylori infection is associated with elevated galactose-deficient iga1 in iga nephropathy. Ren Fail. (2020) 42:539–46. doi: 10.1080/0886022X.2020.1772295. PMID: 32524871 PMC7946026

[136] Wu M-C Leong P-Y Chiou J-Y Chen H-H Huang J-Y Wei J-C . Increased risk of systemic lupus erythematosus in patients with helicobacter pylori infection: A nationwide population-based cohort study. Front Med. (2020) 6:330. doi: 10.3389/fmed.2019.00330. PMID: 32064263 PMC7000519

[137] Wu S Wei F Chen Y Chen Z Luo Y Fan J . Lactiplantibacillus plantarum ZJ316 Alleviates Helicobacter pylori-Induced Intestinal Inflammation by Sustaining Intestinal Homeostasis. Probiotics Antimicrob Proteins. (2025) 17(6):5195–212. doi: 10.1007/s12602-025-10474-w. PMID: 39948232

[138] Magri G Miyajima M Bascones S Mortha A Puga I Cassis L . Innate lymphoid cells integrate stromal and immunological signals to enhance antibody production by splenic marginal zone b cells. Nat Immunol. (2014) 15:354–64. doi: 10.1038/ni.2830. PMID: 24562309 PMC4005806

[139] Jackson-Jones LH Duncan SM Magalhaes MS Campbell SM Maizels RM McSorley HJ . Fat-associated lymphoid clusters control local igm secretion during pleural infection and lung inflammation. Nat Commun. (2016) 7:12651. doi: 10.1038/ncomms12651. PMID: 27582256 PMC5025788

